# Rationale and design of a three-arm randomized clinical trial to improve drug use and retention in care of people with opioid use disorder on buprenorphine: OVERCOME II study

**DOI:** 10.1016/j.cct.2026.108226

**Published:** 2026-01-17

**Authors:** Irene Pericot-Valverde, Moonseong Heo, Kaileigh A. Byrne, Alison Karasz, Angelica Perez, Snehal Lopes, Megan Groome, Sarah Voss, Ashley King, Katy Barnick, Alain H. Litwin

**Affiliations:** a Department of Psychology, College of Behavioral, Social, and Health Sciences, Clemson University, Clemson, SC 29634, USA; b Department of Public Health, College of Behavioral, Social, and Health Sciences, Clemson University, Clemson, SC 29634, USA; c Department of Family Medicine and Community Health, UMass Chan Medical School, Worcester, MA 01655, USA; d Addiction Medicine Center, Department of Medicine Prisma Health-Upstate, Greenville, SC 29605, USA; e Department of Medicine, University of South Carolina School of Medicine, Greenville, SC 29605, USA

**Keywords:** Recovery coach, Opioid use disorder, Buprenorphine, CB4CBT, TAU, Neurophysiology

## Abstract

Medications for opioid use disorder (MOUD) are effective in reducing opioid use, but retention to buprenorphine remains suboptimal among OUD patients. Integration of computer-based cognitive behavioral therapy (CBT4CBT) and utilization of recovery coaches (RCs), called RC + CBT4CBT-Buprenorphine, into OUD care could improve drug use and retention in care among patients taking buprenorphine. The OVERCOME II (**O**UD Inter**ve**ntion **R**ecovery **C**oach CBT and **O**UD **Me**dications II) study is a three-arm 1:1:1 randomized clinical trial designed to test the remotely delivered RC + CBT4CBT-Buprenorphineintervention as an adjunct to buprenorphine treatment for patients with OUD, and to compare it with both CBT4CBT-Buprenorphine alone and the treatment as usual (TAU). Patients (*n* = 90) who have newly initiated buprenorphine in the past 90 days are planned to be recruited. Comprehensive measures on demographics, various conditions/constructs, and outcomes will be collected from medical charts and survey instruments. Additionally, neurophysiological outcomes and neurocognitive inhibitory controls will be measured using the NEXUS-10-MKII physiological system with BioTrace+ software and computerized Drug Go/NoGo Task, respectively. Qualitative analysis of interviews with *N* = 2 RCs and *N* = 15 participants will also be conducted. The study will be the first to examine the effectiveness of the remotely delivered RC + CBT4CBT-Buprenorphine intervention on drug use (during the first 8 weeks and follow-up) and retention in care, as well as on various neurophysiological and cognitive performances in comparison to CBT4CBT-Buprenorphine alone and TAU.

**Clinical Trial Registration:** This study was registered at ClinicalTrials.gov (NCT06102200, www.clinicaltrials.gov/) on August 31, 2023, with a title, An Integrated Intervention Involving Recovery Coaching and Cognitive Behavioral Therapy for Opioid Use Disorder (OVERCOME 2).

## 1. Introduction

Opioid use disorder (OUD) affects an estimated 2.1 million people in the US and is expected to continue to increase. [1] Medications for opioid use disorder (MOUD), including buprenorphine, have been demonstrated to be effective at producing substantial reductions in opioid use and other illicit drugs, risk of overdose, criminal activity, and other risky drug-related behaviors. [2–6] Despite the effectiveness of MOUD, retention to buprenorphine is suboptimal [7] as a recent systematic review indicated that only 57% of individuals who initiate MOUD are retained 6 months later, and 46% in 12 months. [8] In addition, high rates of polysubstance use among people receiving MOUD were associated with lower adherence and retention to MOUD. [9,10] As such, integrating additional therapeutic strategies into MOUD protocols to address these deficits is urgently needed.

Peer recovery coaching is a form of nonclinical, peer support aimed at helping individuals with substance use disorders (SUDs) achieve and maintain recovery. [11] We have developed a recovery coaching assertive community engagement (ACE) model by adapting from the evidence-based model of assertive community treatment (ACT) for persons living with serious mental illnesses. [12–14] Recovery coaches (RCs) are individuals with lived experience with substance use and successful recovery. In addition to their lived experience, recovery coaches receive formal training on substance use coaching following the assertive community engagement (ACE) model and receive ongoing supervision. There is evidence that this RC recovery coaching improves initiation, long-term engagement in the recovery process and reduces substance use. [15–18] Nonetheless, our recent systematic review revealed that recovery coaches have not been shown to improve OUD outcomes beyond MOUD initiation. [19]

Cognitive behavior therapy (CBT) is an integrative approach involving cognition, affect, and environment and emphasizes learning new skills and coping behaviors in order to achieve abstinence and avoid relapse. [20] Although CBT can be delivered by professionals, including social workers, psychiatrists, and psychologists, using various modes of delivery, such as in-person and via computer-based interfaces, a computer-based training version of CBT for SUDs, known as CBT4CBT, has emerged as a promising tool for delivering CBT. [21–24] Nonetheless, a recent systematic review of randomized clinical trials (RCTs) combining computerized CBT and pharmacotherapy showed that the addition of computerized CBT only produces a small, significant effect in drug use, [25] but no effect was seen in the two buprenorphine trials. [26,27] However, a pilot RCT examined a web-based CBT (CBT4CBT-Buprenorphine) training adapted for office-based buprenorphine, and found that those who were randomized to receive CBT4CBT-Buprenorphine submitted more urine samples that were negative for opioids (91.3% vs 63.9%) and for all drugs tested during the treatment than those assigned to buprenorphine alone (81.6% vs. 29.9%). [28] However, CBT4CBT module completion was suboptimal, as participants completed only about half (52.5%) of the intended 8 CBT4CBT modules).

To complement the effect of CBT4CBT, we developed and tested an integrated intervention that combines Recovery Coaching and CBT4CBT-Buprenorphine (RC + CBT4CBT-Buprenorphine), guided by the well-established behavioral change model of Information, Motivation, Behavioral Skills (IMB) model. [29] The IMB model asserts that information, motivation, and behavioral skills are fundamental determinants that influence individuals’ behavior change. The CBT4CBT component will provide information related to substance use (several modules, which includes understanding drug use, patterns of substance use, triggers) and adherence to buprenorphine (module on buprenorphine, which includes an introduction to buprenorphine, what to expect in treatment, handling common problems, and talking to family members about OUD and buprenorphine, and encouraging patients to adhere to their treatment program and stay on buprenorphine). The Recovery Coach component will enhance motivation. Finally, both components, CBT4CBT and Recovery Coach, will improve behavioral skills in that the CBT4CBT program will teach the skills, whereas the recovery coach will provide role modeling and reinforce skills, encourage the participant to practice skills, and promote completion of modules and homework, thereby leading to a reduction of drug use and improved retention in buprenorphine treatment. Our group found that adding an RC to CBT4CBT-Buprenorphine improved adherence to the CBT modules and improved drug use outcomes compared to treatment as usual (TAU) with buprenorphine alone. [30]

The critical gap, however, is that we did not compare the integrated recovery coach intervention RC + CBT4CBT-Buprenorphine to CBT4CBT-Buprenorphine alone nor assess retention in care. Therefore, the additional benefit of adding a recovery coach to CBT4CBT-Buprenorphine is unknown. An additional research gap is the examination of craving and physiological arousal in response to drug cues, as well as excessive responsiveness to negative events, which may contribute to the low rates of adherence and recovery among individuals receiving MOUD. [31,32] A recent RCT [33] demonstrated that the CBT4CBT intervention showed a trend towards reduction in attentional bias for drug, which suggests that high levels of engagement with CBT4CBT have the potential to improve cognitive control over drug cues and triggers. However, engagement with this intervention is key to such improvements in cognitive control; therefore, integrating recovery coaches into the intervention may represent an effective way to enhance engagement with CBT4CBT. Building on this plausible mechanism, we attempt to assess the effect of RC + CBT4CBT-Buprenorphine on inhibitory control over drug cues using the Drug Go/NoGo Task adapted for opiate cues in individuals with opioid use disorder.

To fill those gaps, the present OVERCOME II (**O**UD Inter**ve**ntion **R**ecovery **C**oach CBT and **O**UD **Me**dications II) randomized clinical trial study proposes to test the efficacy of RC + CBT4CBT-Buprenorphine for reducing nonprescribed substance use and improving buprenorphine retention compared to TAU and CBT4CBT-Buprenorphine alone (Aim 1), to explore neurocognitive and physiological mechanisms of action of the integrated intervention (Aim 2), and to qualitatively evaluate our RC + CBT4CBT-Buprenorphine intervention to be able to refine the treatment intervention (Aim 3).

## 2. Methods

### 2.1. Setting

The study will be conducted at the Prisma Health Recovery Program (PHRP) in Greenville, South Carolina. This office-based buprenorphine program is staffed by one part-time physician, two full-time nurse practitioners, two full-time addiction registered nurses, one full-time substance use counselor, and one part-time social worker. We have access to part-time psychiatrists (1.5 days each week staffed by 3rd psychiatry residents). The OVERVOME II study is built upon a pilot OVERCOME I study that compared outcomes only between TAU and RC + CBT4CBT-Buprenorphine. [30] The OVERVOME II study is funded by the National Institute on Drug Abuse (NIDA) as a project of the National Institutes of Health (NIH) HEAL initiatives, approved by the Prisma Health IRB (1930664), and registered in the ClinicalTrials.gov (NCT06102200).

### 2.2. Study arms and interventions

#### Treatment as usual (buprenorphine only) arm:

TAU consists of weekly, bi-weekly or monthly visits (at the discretion of the provider) in-person or virtually at an outpatient buprenorphine clinic. Beyond the prescription of buprenorphine, psychosocial support will be provided as the standard of care in the clinic. When a patient needs additional support, the provider schedules an appointment with a counselor.

#### CBT4CBT-Buprenorphine alone arm:

Participants will be provided with the 8-session (module) self- directed CBT4CBT-Buprenorphine system for teaching with one module on the basics of buprenorphine and 7 CBT core skills tailored around issues related to buprenorphine and OUD and other SUDs: (1) introduction to functional analysis of substance use; (2) strategies for recognizing and coping with craving; (3) refusal skills and assertiveness; (4) training in problem solving skills; (5) strategies for recognizing and changing thoughts; (6) decision making skills; (7) how to use CBT skills to reduce HIV/HCV risk. Each module takes 30 min to complete and includes on-screen narration, animation, quizzes, and interactive exercises to teach and model effective use of skills. Modules end with a practice exercise. Study coordinators will instruct participants about the proper use of the CBT4CBT-Buprenorphine system at baseline.

#### Recovery Coach + CBT4CBT-Buprenorphine arm:

This intervention follows the ACE model with approaches to provide a holistic, person-centered, and strength-based support in four big areas: emotional/motivational, informational, instrumental, and affiliational areas. [34] (Table 1) Core characteristics of the ACE model include the team approach (RC integrated with the buprenorphine recovery program team, including addiction medicine clinicians, registered nurses, social workers, and substance use counselors). Recovery coaching services are provided where patients live and work: personalized care; continuous care with trusted team members; flexible care; comprehensive care; and services when they are needed. FAVOR-Greenville has developed and continually maintains a 20-page community directory of resources. RCs are trained as both Certified Peer Support Specialists and Certified Assertive Community Engagement Specialists under the National Association of Alcohol and Drug Abuse Counselors. In addition to formal training, they are required to have 1 year or more in recovery and adhere to the FAVOR code of conduct. Two hired Prisma Health recovery coaches were trained in the use of CBT4CBT-Buprenorphine by an expert in CBT and received ongoing supervision to ensure adherence to the study protocol.

The first encounter with the recovery coach will occur at the baseline (RC + CBT4CBT-Buprenorphine arm only) after participants are deemed eligible and have completed all research assessments. During this first encounter, the recovery coach will perform a needs assessment using recovery capital matrix, based on which the recovery capital matrix, dyad of RC and participant will discuss a customized recovery plan for 12 weeks, always starting with the capital domain that requires more immediate attention in addition to explaining the 8-week CBT4BT-Buprenorphine program and instructing the participants to start completing the modules. RCs and participants will meet once a week, virtually or in person, where they will discuss the CBT4CBT-Buprenorphine module completed, resolve questions and doubts, complete practice exercises, and help with the homework. Recovery coaching services will be delivered via Zoom calls, phone calls, and text messages, and required to initiate at least one meaningful contact (video Zoom) and 3 check-ins (i.e., text message, email, and/or quick call) per week. In addition, the RCs will assess the status of participants’ recovery capital and move towards the next capital needing attention, and will have access to funds capped at $500 per participant to be used at their discretion for providing personalized support services to participants during the 8-week treatment period. The RC will assume responsibility for the ongoing therapeutic relationship. All RCs will be supervised by a senior recovery coach on a weekly basis throughout the course of the treatment.

### 2.3. Participants defined by inclusion and exclusion criteria

This study plans to recruit a total of *N* = 90 participants with OUD receiving buprenorphine. Inclusion criteria for participating in this study include: (1) adults 18 years or older; (2) DSM-V diagnosis of OUD; (3) currently receiving sublingual buprenorphine/naloxone and/or buprenorphine; (4) have initiated buprenorphine within last 90 days from baseline; (5) willing to accept a random assignment to either standard of care, CBT4CBT-Buprenorphine or RC + CBT4CBT-Buprenorphine; and (6) having adequate computer skills (verified using computer literacy survey). Exclusion criteria: (1) having a severe medical or psychiatric disability that could impair ability to perform study-related activities (determined by the clinician); (2) being unable to independently read and/or comprehend the consent form or other study materials; (3) being unable to read/speak English; and (4) having current suicidal ideation based on the Patient Health Questionnaire-9.

### 2.4. Recruitment process

Participants will be recruited (brochures, active referrals from clinicians, research coordinators approaching patients in the clinic, and participant referrals) Potential eligible patients will first be identified through medical chart reviews. Eligible participants who express interest in learning about our study will have a discussion with a research coordinator. Prior to the administration of any study measures, the research coordinator will obtain verbal consent for screening, which briefly describes the study and reinforces the confidentiality of all survey information. Interested patients who are eligible for the study after the initial screen will sign a written informed consent document.

### 2.5. Assessment schedules and incentives

Research assessments will be completed at baseline (randomization), during weekly visits 1–8 of treatment, and then at follow-up assessments 1, 3, and 6 months after the completion of the treatment period. Major research visits (baseline, end of treatment, and follow-ups) will be conducted in person, while all other weekly research visits (i.e., 1–7) will be conducted virtually or in person, based on participant preference. During visits, participants will complete various research materials, including questionnaires, clinical interviews, and saliva drug tests. Participants will self-administer the saliva drug tests while observed virtually by the research coordinator. Participants will be compensated $40 for the baseline session, $20 for visits weeks 1–7, and $40 for week 8 (i.e., end of treatment) and three follow-ups. Participants will receive $50 for the first neuro laboratory session and $75 for the second one. Finally, participants involved in Aim 3 (*N* = 15) will be compensated $25 for participating in the interview. The maximum amount of compensation will be $465/$490.

### 2.6. Randomization, concealment, blinding and data management

The participants will be randomized in a 1:1:1 ratio with varying masked block sizes to equally distribute *N* = 30 participants to each arm… The random allocation will be concealed, passcode-protected and stored in a secure Research Electronic Data Capture (REDCap) database. Only designated study coordinators will have access to the random allocation code and will dispense upon the availability of patients eligible for enrollment and randomization. Due to the open-label nature of the interventions, it is not feasible to blind the participants or the research coordinators duing assessments conducted at the research visits. However, we will use standardized treatment manuals and protocols to ensure the consistent delivery of the intervention and study assessments across participants, thereby maintaining internal validity of our study and minimizing the risk of potential bias due to the unblinding.

**Fig. 1.**
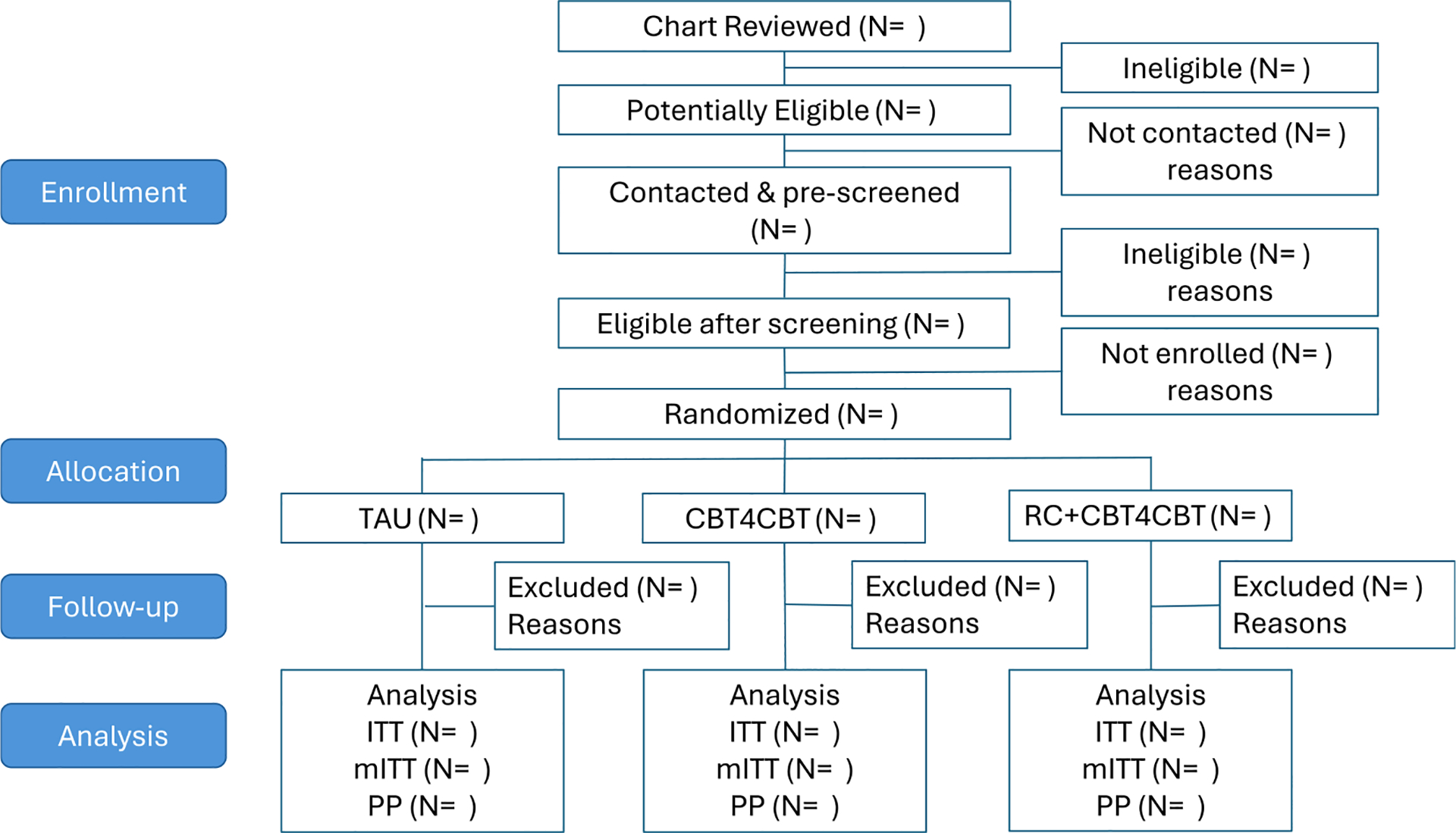
Tentative schematic CONSORT flow chart.

The REDCap database will also be used to collect and manage data, ensuring that all data will be entered into REDCap with no personal identifiers, and source documents will identify participants by study ID. Access to computers used for data entry, management, and analysis will be passwordprotected and limited to the study coordinators. Personal identifying information (including locator information) will be kept in a secure area and a locked file cabinet. More details will be described and specified in a separate data management plan (DMP) document following the HEAL-initiative data ecosystem guidelines. A tentative schematic CONSORT diagram depicting the entire study flow from enrollment steps to the analysis phase is presented in Fig. 1.

### 2.7. Measures

All study measures for primary, secondary, and exploratory outcomes will be assessed using survey instruments and medical charts listed in Table 2 along with purposes and assessment time frames. Baseline demographic characteristics will include age, gender, ethnicity, race, sexual orientation, education, marital status, annual income, housing status, employment status, handedness (right- or left-handed), and method of transportation. Baseline disease characteristics will include hepatitis C infection, HIV infection, and comorbidities that include diabetes, liver disease, renal disease, and chronic pulmonary diseases. Retention in care will be assessed based on medical chart data on buprenorphine prescription records. All measures in Table 2, except for those specific to RC or CBT4CBT activities, will be assessed for all participants. The primary outcome for aim 1 will be longitudinal weekly positive responses for any drug use ascertained by saliva toxicology test during the 8-week study period. To this end, the study will use a 14-panel saliva drug test (Lochness Medical Supplies, Inc.) that screens for alcohol, amphetamine, barbiturate, buprenorphine, benzodiazepine, cocaine, fentanyl, MDMA, methadone, methamphetamine, opiate, oxycodone, THC/Cannabinoids, and phencyclidine (PCP). A secondary outcome will be retention in care, to MOUD in particular, where MOUD includes buprenorphine, methadone, or extended-release naltrexone.

For the aim 2 examinations, participants will complete laboratory sessions twice, once at baseline and again after completing the initial 8-week period, in between which the study will measure changes in neurophysiological outcomes, cognitive, and self-report outcomes. The laboratory sessions will be conducted on days separate from those of other assessments, both at baseline and at the completion of treatment, to mitigate participant burdens. Neurophysiological outcomes will be measured using the NEXUS-10-MKII physiological system with BioTrace+ software and include electroencephalogram (EEG), skin conductance level (SCL), heart rate variability (HRV), and respiration rate (RR) during the cue reactivity task, which will be the primary outcomes for the aim 2 examinations. Neurocognitive outcomes will be assessed using a novel computerized Drug Go/NoGo Task, designed to measure inhibitory control over drug-related stimuli. Participants will be instructed to press a key when neutral Go cues are presented (e.g., candy) and withhold their responses when drug-related NoGo cues (e.g., opioid pills) are shown. The task uses a block-design pictorial paradigm involving 400 trials, with 15 images per 75-s block (5 s per image) and interleaved 15-s black screens. Approximately 75% of the stimuli will be neutral Go stimuli, and 80% will be drug-related NoGo stimuli. The primary outcome measure is the number of commission (NoGo) errors. During the Go/NoGo task, event-related potentials (ERPs) will be recorded. Components of interest include N200, P300, and error-related negativity (ERN), which will be extracted for both stimulus (NoGo cues) and response-locked neural activity using EEGLAB. [35–37] Self-report outcomes include craving and coping skills using the 3-item Opioid Craving Scale [38] and the Coping Strategies Scale. [39]

For the aim 3 qualitative examinations, the study will conduct individual qualitative interviews with the recovery coaches (*N* = 2) and participants enrolled in the RC + CBT4CBT- Buprenorphine intervention (*N* = 15). Interviewers will emphasize and assess: (1) barriers and facilitators to implementation, (2) perceived weaknesses, strengths, rewards, and burdens of the intervention, and (3) recommended changes to improve the intervention.

### 2.8. Aim-by-aim statistical analytic plans

A detailed statistical analysis plan (SAP) will be documented in a separate file, and thus, here we briefly describe analysis plans. All analyses will be performed using SAS v9.4. (SAS inc, Cary, NC, USA) and R software.

#### Aim 1 analysis plan:

The primary analytic sample will include all randomized participants, referred to as the intention-to-treat (ITT) sample. For the analysis of data from this ITT sample, the group membership will remain unchanged as randomized, even if participants cross over to the other groups during the trial. We will also conduct modified ITT (mITT) analysis, including only participants in the intervention arms who opened at least one CBT4CBT module, and per-protocol (PP) analysis, including those who completed 50% or more modules and/or those who engaged with a RC at least once. To compare the longitudinal primary binary outcome of drug-free test results across the three groups, we will apply generalized mixed-effects linear models to the ITT sample. The primary comparisons will be conducted in pairwise comparisons with a Bonferroni-corrected two-tailed significance level of 0.017 = 0.05/3: 1) RC + CBT4CBT-Buprenorphine vs. CBT4CBT-Buprenorphine; 2) RC + CBT4CBT-Buprenorphine vs. standard of care; and 3) CBT4CBT-Buprenorphine vs. standard of care. As this is an RCT with ITT sample, no adjusting variables will be included in the model. The analysis of secondary outcomes, such as retention in care during the study and follow-up periods, will be similarly conducted.

#### Aim 2 analysis plan:

First, to test whether changes from baseline to the end of treatment in scores of neurophysiological responses (SCL, respiration, and HRV), Drug Go/NoGo Task commission errors, coping strategies, self-reported craving, and are different between RC + CBT4CBT-Buprenorphine and other groups in a pairwise comparison manner, we will use *t*-test or Wilcoxon rank sum test (depending on the results of normality test). If adjustment for covariates is deemed necessary, we will apply analysis of covariance (ANCOVA). Second, to test whether outcome scores themselves are different, overall or by group, between baseline and the end of the first 8 weeks, we will use a paired t-test or Wilcoxon signed-rank sum test. Lastly, correlation analysis will be used to explore the association among physiological responses, treatment engagement indicators (i.e., number of modules completed, number of CBT4CBT homework assignments completed, and number of contacts with the recovery coach) and Drug Go/NoGo Task (commission errors), coping strategies, self-reported craving, and ERP components (N200, P300, ERN).

#### Aim 3 analysis plan:

Interviewers’ transcriptions will be uploaded into NVIVO, a qualitative data analysis program that facilitates the rapid organization and retrieval of thematic data. The qualitative analysis team will develop a preliminary coding scheme. Preliminary codes will include barriers and facilitators of implementation; perceptions of strengths, weaknesses, rewards, and burdens; and recommendations to improve the intervention. The initial coding scheme will be applied to a subset of data, and will be subsequently revised and reapplied to the data in an iterative fashion until it is deemed sufficiently parsimonious and comprehensive.

### 2.9. Handling missing data

Every effort will be made to limit the amount of missing primary outcome data in this study. Although the primary analytic strategy involves analyzing available data using mixed-effects models, which is unbiased under missing at random (MAR) assumptions, we will conservatively assume in a sensitivity analysis that missing saliva samples will be considered drug-positive based on the current literature, [23,26,28] that is, the worst-case scenario imputation. In addition, we will also apply last-observation-carried-forward (LOCF) imputation as well as fully specific conditional specification multiple imputation methods, [40] which are applicable to non-ignorable missing data. Characteristics of patients who are lost to follow-up will be compared to those who complete the study to assess the degree of any selection bias due to attrition.

### 2.10. Power analysis

The power analysis computed the minimally detectable effect size for the aim 1 primary outcome, longitudinal weekly positive saliva drug test response, with the ITT sample after consideration of 10% attrition without completing all 8 post-baseline tests during the trial resulting in *N* = 27 per group. A conservative power analysis is conducted based on this sample size although observations from lost follow-up participants will be included in the primary statistical analysis. With a two-tailed Bonferroni-corrected 0.017 significance level and intra-class correlation coefficient (icc) of the 8 post-baseline repeatedly measured binary outcomes within participants assumed to be less than 0.1, any pairs of the groups (or, especially between TAU and RC + CBT4CBT-Buprenorphine intervention) with >20% outcome difference (e.g., 38% vs. 58%) will be detected with >80% statistical power by the generalized mixed-effects linear models.

## 3. Results

The first enrollment was made in October 2023, and follow-up data collection is still ongoing as of May 2025 although recruitment and randomizations of *N* = 90 participants (*N* = 30 in each arm) have been completed after excluding 2 randomized participants who were identified as ineligible after randomization. The two randomized patients were identified by study coordinators as having insufficient computer skills to receive the web-based CBT4CBT during the study, despite their self-reported adequacy during the screening phase. We determined this as a serious protocol violation and randomly assigned two additional participants to the interventions that were formerly assigned to the two excluded participants who violated the protocol inclusion criteria. The pace of recruitment was approximately five participants per month in a single office-based clinic, suggesting that recruitment of participants from multiple sites across different settings would be necessary for a larger-scale trial.

## 4. Discussion

The OVERCOME II study has several innovative features that have not been implemented in any prior studies. First, it will be among the first studies to examine the effectiveness of recovery coaching that will be delivered remotely using computer or smartphone platforms for improving retention to MOUD among individuals with OUD. Although earlier studies explored the impact of incorporating recovery coaching on MOUD initiation rates, this will be the first study to examine the impact of recovery coaching for improving retention among patients already enrolled on MOUD, yet have newly initiated within the past 90 days.

Second, it will also be the first study to test the effectiveness of an intervention combining recovery coaching and CBT4CBT-Buprenorphine in comparison to CBT4CBT-Buprenorphine alone, a comparison that has not been studied. Prior RCTs assessing the effectiveness of recovery coaching for treating SUDs have combined recovery coaching with a variety of therapeutic strategies, including intensive referral interventions, skills training, and multicomponent interventions (involving case management, individual/group treatment, and other services). In addition, the OVERCOME II study is the first RCT examining whether an integrated RC + CBT4CBT-Buprenorphine intervention influences inhibitory control and craving. Thus, we uniquely test whether this integrated intervention can improve substance use recovery by reducing craving and improving inhibitory control over such cravings, which provides a potential mechanism of action for the intervention. This RCT will include novel neurocognitive measures to assess changes in neurocognition such as the Drug Go/NoGo inhibition task with tailored opiate NoGo stimuli and neurocognitive event-related potential (ERP) outcomes.

This study will be the largest RCT (*N* = 90) to examine the effectiveness of web-based CBT4CBT-Buprenorphine intervention for patients on buprenorphine. In addition, the majority of the population will have polysubstance use, including cocaine and methamphetamine use, which reflects the reality of OUD patients being treated in office-based buprenorphine programs. In contrast, the Shi et al. study [28] enrolled 20 patients, and excluded patients with current cocaine, benzodiazepine, and alcohol use disorder.

This study is designed to utilize RCs rather than social workers. RCs assist people with SUDs in navigating various recovery pathways and providing personalized, peer-guided guidance. Unlike social workers, RCs have lived experience with addiction and recovery, which enables them to offer a unique set of comprehensive services that are not part of standard social work practice. In addition, recovery coaches have multiple roles, including advocacy, mentoring/role modeling, social support, instrumental support, motivational interviewing, and assistance in navigating rehabilitation programs. The recovery coach offers the patient with flexibility in developing and formulating a plan that suits their specific needs. Recovery coaches provide lived experience, flexible long-term support, additional areas of support (affiliational and emotional), and work within the community.

Participants in this study may feel burdened by the assessments and visits, which appear to be somewhat lengthy and frequent. To mitigate these potential burdens, we will implement several strategies. First, we will maximize efficiency by combining multiple tasks into a single visit whenever possible (i.e., self-reported and saliva tox assessments). Furthermore, neurocognitive tests will be conducted on separate days. We will schedule research visits to coincide with clinical appointments, thereby minimizing the total number of visits. We will offer convenient scheduling options, including early and evening appointments, as well as remote or hybrid weekly visits via Zoom, making it easier for participants to participate in the study. The therapeutic components, including CBT4CBT and related homework, will be conducted remotely, and patients will complete these activities at their convenience. Participants assigned to the RC + CBT4CBT will be scheduled to meet with the coaches either in person or remotely, based on participant preference, and these meetings will be arranged according to the participant’s availability. Overall, the intervention was designed to be easily integrated into adults’ everyday lives, aiming to minimize burden.

## 5. Conclusion

The innovative OVERCOME II study will inform preliminary efficacy of RC + CBT4CBT-Buprenorphine, including effect sizes for drug use (during 1st 8 weeks and follow-up) and retention in care. The Recovery Coach+CBT4CBT-Buprenorphone intervention will be refined and finalized based on the qualitative interviews. If proven effective and feasible to implement in broader settings, a future confirmatory larger-scale study will propose a more comprehensive RCT of a 2-by-2 factorial experimental design with adequate power to test efficacy of RC (yes, no) vs. CBT4CBT-Buprenorphine (yes, no) on improving drug use and retention in care where the (no, no) combination represents TAU.

## Figures and Tables

**Table 1:** Structured services provided by recovery coaches.

Areas	Description	Examples of services provided
Emotional	Demonstrate empathy, caring, and concern in order to improve patient’s self-esteem and confidence	Peer mentoring Listening to problems
Informational	Share knowledge and information; provide life or vocational skills training	Life-skills class (parenting, job interview training) Legal contact, information on multiple routes to recovery, contacts for mental health resources
Instrumental	Provide concrete assistance with individual’s basic needs including social determinants of health	Food availability (food banks, stamps) Housing/shelter Transportation
Affiliational	Facilitate connection with other people to promote social/recreational skills, create community, and acquire a sense of belonging	Recovery community Sports league participation Engagement centers Social/recreational activities Cultural activities Faith-based recovery groups

**Table 2:** Study measures and timeframes.

Instrument	Purpose/Domain	BL*	NB*	W1-W3	W4	W5 - W7	EOT*	NEOT*	1MF - 6MF*
**Demographics**	Sample Characteristics	x							
**Behavioral and clinical factors**									
ASI-Lite Shorten Version	Substance Use Treatment & Counseling	x					x		
AUDIT-C	Alcohol Misuse	x					x		
Tobacco Use	Cigarette and e-cigarette use	x					x		
	CO measurement	x					x		x
Treatment History - Abbreviated	Counseling, Recovery Coaching Services, Psychiatric Services	x					x		x
	Treatment Services Review								x
IBM Model Information Motivation Behavioral Skills	Knowledge Questionnaire	x			x		x		x
	Readiness Ruler	x			x		x		x
	Web CBT Coping Strategies Inventory	x			x		x		x
Behavioral Risk Assessment	Overdose, HIV/HCV High-risk behaviors	x					x		x
PHQ-9	Depressive Symptoms	x					x		x
GAD-7	Anxiety Symptoms	x					x		x
EQ-5D-3L	Quality of Life	x					x		x
Recovery Capital Matrix	Social Determinants of Health - Recovery Capital	x		x	x	x	x		x
**Aim 1 outcomes for testing the efficacy of RC + CBT4CBT-Buprenorphine intervention on drug use and retention**									
Saliva Drug Test	Recent Drug Use	x	x	x	x	x	x	x	x
30 Day TLFB	Substance Use	x							x
7 Day TLFB	Substance Use			x	x	x	x		
Substance Use Treatment Retention	Medication retention assessed via EMR or PDMP	x		x	x	x	x		x
M-MASRI	Buprenorphine Adherence	x		x	x	x	x		x
CBT4CBT Modules	Sessions & Homework Completed			x	x	x	x		
**Aim 2 outcomes on assessments of neurophysiological and cognitive performances**									
No-Go Task	Neurophysiological Response to impulse		x					x	
Cue Reactivity	Neurophysiological Response to Triggers		x					x	
Emotional Regulation Questionnaire	Neuro session assessment emotional response to images		x					x	
Opioid Craving Scale	Research Coordinator Assessed - Craving	x		x	x	x	x		x
	Neuro Session - Craving		x					x	
Delay Discounting	Behavioral Economics	x							
Demand Task	Behavioral Economics	x			x		x		x
**Aim 3 outcomes on feasibility assessment for refinement of the RC + CBT4CBT-Buprenorphine intervention**									
Participant Feedback Form (RC)	Satisfaction with the intervention						x		
Participant Feedback Form (CBT4CBT only)	Satisfaction with the intervention						x		
Session Checklist	Fidelity to protocol			x	x	x	x		
Working Alliance Index	Alliance with RC						x		
Recovery Coach Tracking Form	Interactions between study participants and the RC	x		x	x	x	x		
AE/SAE Tracking	Assessing adverse events reported by patients or in EMR			x	x	x	x	x	x

Note:

* in-person visits.

Abbreviations: AE: Adverse Event; ASI: Addiction Severity Index; AUDIT: Alcohol Use Disorder Identification Test; BL: Baseline; CBT: Cognitive Behavior Therapy; CO: Carbone Monoxide; EMR: Electronic Medical Record; EOT: End of Treatment; GAD: Generalized Anxiety Inventory; IBM: Information Motivation Behavioral; MF: Month Follow-up; M-MASRI: Modified Medication Adherence Self-Report Inventory; NBL: Neuro Baseline; NEOT: Neuro End of Treatment; PDMP: Prescription Drug Monitoring Program; PHQ: Patient Health Questionnaire; RC: Recovery Coach; SAE: Serious Adverse Event; TLFB: Timeline Follow-Back; W: Week.

## Data Availability

All data generated or analyzed for this report are included in this article.

## Abbreviations:

ACE: Assertive Community Engagement
ACT: Assertive Community Treatment
ANCOVA: Analysis of Covariance
CBT: Cognitive Behavior Therapy
CONSORT: Consolidated Standards of Reporting Trials
DMP: Data Management Plan
DSM-V: Diagnostic and Statistical Manual of Mental Disorders Fifth Edition
ED: Emergency Department
EEG: Electroencephalogram
ERN: Error-Related Negativity
ERP: Event-Related Potential
FAVOR: Faces and Voices of Recovery
HCV: Hepatitis C Virus
HEAL: The Helping to End Addiction Long-term
HIV: Human Immunodeficiency Viruses
HRV: Heart Rate Variability
ITT: Intention-To-Treat
mITT: modified Intention-To-Treat
MOUD: Medications for Opioid Use Disorder
NC: North Carolina
NIDA: The National Institute of Drug Abuse
NIH: The National Institutes of Health
OUD: Opioid Use Disorder
OVERCOME II: OUD Intervention Recovery Coach CBT and OUD Medications II
PHRP: Prisma Health Recovery Program
PP: Per-Protocol
RC: Recovery Coach
REDCap: Research Electronic Data Capture
SAP: Statistical Analysis Plan
SAS: Statistical Analysis System
SCL: Skin Conductance Level
SUD: Substance Use Disorder
TAU: Treatment As Usual
USA: United States of America
